# High-Risk Indicators of Renal Involvement in Primary Sjogren's Syndrome: A Clinical Study of 1002 Cases

**DOI:** 10.1155/2019/3952392

**Published:** 2019-02-17

**Authors:** Jing Luo, Yu-Wei Huo, Jian-Wu Wang, Hui Guo

**Affiliations:** ^1^Division of Rheumatology, Department of Medicine, The Second Hospital of Shanxi Medical University, Taiyuan, Shanxi 030001, China; ^2^Division of Nephrology, Department of Medicine, The Second Hospital of Shanxi Medical University, Taiyuan, Shanxi 030001, China; ^3^Division of Nephrology, Department of Medicine, Shenzhen University General Hospital, Shenzhen, Guangdong 518005, China

## Abstract

**Objective:**

A retrospective analysis of clinical characteristics and immunological manifestations of primary Sjogren's syndrome (pSS) patients with or without renal involvement was conducted in order to elucidate the potential risk factors of renal damage in pSS and evaluate the condition.

**Methods:**

A total of 1002 patients, who fulfilled the 2002 classification criteria for pSS from the Second Affiliated Hospital of Shanxi Medical University, were enrolled in the cross-sectional study. Clinical, immunological, and histological characteristics were compared between pSS patients with and without renal involvement, and potential risk factors of renal involvements in pSS patients were examined by multivariate analysis.

**Results:**

Among these pSS patients, there were 162 cases (16.17%) with and 840 cases (83.83%) without renal damage. Serious edema of both lower limbs, interstitial nephritis, and renal tubular acidosis were found in the pSS with renal damage group. Compared with simple pSS patients, the levels of creatinine, cystatin C, and alpha-1-microglobulin (*α*_1_-MG) in the pSS with renal damage group were significantly increased. The difference between the two groups was statistically significant (*P* < 0.05). The AUC of the combination of creatinine and *α*_1_-MG and creatinine, *α*_1_-MG, and creatinine was statistically larger than that of creatinine, and the biomarker of the biggest AUC is the combination of creatinine and *α*_1_-MG.

**Conclusion:**

The main clinical manifestations of pSS with renal damage were edema of the lower limbs, interstitial nephritis, and renal tubular acidosis. Creatinine and *α*_1_-MG are effective indicators for renal function in pSS, which may provide a better understanding for clinical decision-making.

## 1. Introduction

Sjogren's syndrome (SS) is a chronic progressive autoimmune disorder characterized by lymphocytic infiltration of the exocrine glands, which affects the salivary and lacrimal glands, presenting dryness of the mouth and eyes. The majority of infiltrating mononuclear cells are CD4+ T cells [1]. Some patients may present diverse extraglandular impairment such as that in the lungs, kidneys, nervous system, and skin affected by this disorder [2]. The predominant serologic findings of pSS are positive anti-nuclear antibodies (ANA), anti-SSA antibodies, and anti-SSB antibodies. Renal involvement is easily ignored by the physicians because the clinical symptoms are often insidious. Growing evidence suggests that patients with pSS may have greater renal injury risk than the general population and the most common renal disease in SS is tubulointerstitial nephritis, responsible for renal tubular acidosis in 20% [3]. However, it is still challenging to diagnose renal involvement in pSS patients.

In the present study, we described the clinical presentation and serologic findings of 840 patients with pSS without renal involvement and 162 patients with renal involvement. We also analyzed whether biochemical markers were useful in identifying renal disease in pSS patients to guide further clinical work.

## 2. Materials and Methods

### 2.1. Methods

#### 2.1.1. Study Population and Clinical Data

A total of 1002 patients who fulfilled the 2002 classification criteria [4] for pSS from the Rheumatology Department of the Second Affiliated Hospital of Shanxi Medical University between September 2013 and September 2017 were enrolled in this study. The study was approved by the Ethical Committee of the Second Affiliated Hospital of Shanxi Medical University (approval # 2016KY007). The study design conformed to the current National Health and Family Planning Commission of China ethical standards, with written informed consent provided by all patients.

Sjogren's syndrome without other autoimmune diseases is called pSS. pSS patients were diagnosed with clinical data as oral and ocular dryness, constitutional symptoms, vasculitis, and joint, skin, pulmonary, kidney, gastrointestinal tract, and endocrine involvement. The clinical observation items included age, gender, course of disease, glandular symptoms (xerostomia and xerophthalmia), and extraglandular symptoms (arthritis, erythema, edema, and digestive, respiratory, and renal involvement). Routine laboratory examinations were performed including routine blood test, routine urine test, liver function examination, nephric function examination, erythrocyte sedimentation rate (ESR), cystatin C, and *α*_1_-MG. Biochemical tests were performed using standard methods in a Beckman Coulter AU 5800 chemistry analyzer, and serum creatinine measurements were used by an IDMS-traceable method. Immunologic examinations which included anti-SSA, anti-SSB, and rheumatoid factors were performed using an immunoblotting method.

#### 2.1.2. Assessment of Renal System Involvement

We identified those with clinically significant renal involvement.

Clinically significant renal involvement in pSS, either interstitial nephritis or GN, was defined by one or more of the following criteria:

1. Renal tubular acidosis (RTA). Subtypes of RTA were determined as follows [5]: RTA type I (distal): hyperchloremic acidosis with a minimum urine pH ≥ 5.3 and low/normal plasma potassium (<5.5 mmol/L), based on reduced H+ secretion in the distal tubule; RTA type II (proximal): hyperchloremic acidosis with a minimum urine pH < 5.3 and low/normal plasma potassium (<5.5 mmol/L), based on reduced HCO3− reabsorption in the proximal tubule; and RTA type IV: hyperchloremic acidosis with a minimum urine pH < 5.3 and high plasma potassium (≥5.5 mmol/L), based on reduced H+ and K+ excretion in the distal tubule
2. Kidney biopsy demonstrating histologic features compatible with glomerulonephritis, interstitial nephritis, or both
3. Fanconi syndrome not associated with any known cause
4. Elevated serum creatinine levels
5. Proteinuria > 500 mg/24 hours
6. Active urine sediment (>3 red blood cells per high-power field or red blood cell casts)

### 2.2. Statistical Analysis

Normally distributed variables were expressed as mean ± standard deviation (SD) and compared using independent samples *t*-test or one-way ANOVA. Nonparametric variables were expressed as medians and interquartile range (IQR) and compared using Mann–Whitney *U* or Kruskal–Wallis test. Categorical variables were compared using a *χ*2-test. To examine correlations between risk factors and renal involvement, univariate analyses were used, firstly based on biological plausibility and literature review. Variables with *P* < 0.05 in univariate analysis were then included in a multivariate analysis using logistic regression. Statistical significance was set at *P* < 0.05. All analyses were conducted using SPSS 22.0 statistical software packages. Receiver operating characteristic (ROC) curves were plotted to explore the significance of multiple biomarkers for renal function in pSS. The differences among the areas under the receiver operating characteristic (ROC) curves (AUC) were calculated by MedCalc Software (version 15.2.0; MedCalc Software, Belgium).

## 3. Results

### 3.1. The Characteristics of pSS Patients with or without Renal Involvement

Demographic, clinical, histological, immunological, inflammatory feature, and outcome measure data were presented in Tables 1 and 2, collected from -162 pSS patients with and 840 without renal involvement. The female to male ratio in pSS patients is 779 : 61 (92.7%). Most patients presented to the hospital at 49 years old for the first interview, and an average disease course was approximately 5 years. Compared with pSS patients without renal involvement, those with renal involvement showed much higher levels of prealbumin, anti-scl-70, rheumatoid factor (RF), anti-extractable nuclear antigen (anti-ENA), anti-SSA, anti-SSB, anti-SM, globulin, urea nitrogen, cystatin C, creatinine, *α*_1_-MG, serum *β*2 microglobulin (*β*_2_-MG), uric acid, Cl, lipoprotein-a, acid phosphatase, ESR, parathyroid hormone (PTH), and carcinoembryonic antigen (CEA), but reduced level of monocyte, anti-SSA, total protein, albumin, carbon dioxide combining power (CO2CP), Ca, red blood cell (RBC), hemoglobin (Hb), apolipoprotein-A_1_, immunoglobulin M (IgM), and complement-C3 (*P* < 0.05). Comparison of the two groups of clinical manifestations is shown in Tables 1 and 2.

### 3.2. The Characteristics of Renal Involvement in Primary Sjogren's Syndrome Patients

The SS patients with renal involvements showed glandular symptoms (xerostomia and xerophthalmia) and extraglandular symptoms (arthritis, erythema, edema, and digestive, respiratory, and renal involvement). Pathological features of patients with pSS with renal involvement are shown in Table 3. In the 12 biopsy patients with pSS with renal involvement, 6 cases had interstitial nephritis and 3 cases had mesangial glomerulonephritis. Three cases had membranous glomerulonephritis, one case diabetic nephropathy, and one case IgA nephropathy.

And the renal damage is shown in Table 4. The prevalence of edema of both lower limbs was higher than 20%. Meanwhile, the occurrences of hypourocrinia, frequent micturition, urgency of urine, hematuria, and diuresis were comparatively low.

### 3.3. Specific Factors Associated with Renal Involvement in pSS Patients

A series of indicators commonly used in clinical practice were selected first by univariate analysis and then logistic regression analysis as potential risk factors for renal involvement in pSS. As is shown in Tables 4 and 5, a series of variables were found to be associated with renal involvement. Compared with pSS patients without renal involvement, edema of both lower limbs and digestive tract involvement were important clinical manifestations (*P* < 0.05).

There was a statistical significance in creatinine, cystatin C, *α*_1_-MG, and chloridion between pSS patients with and without renal damage.

### 3.4. Comparison of ROC Curves and AUC of Creatinine, Cystatin C, and *α*_1_-MG

**Figure 1:**
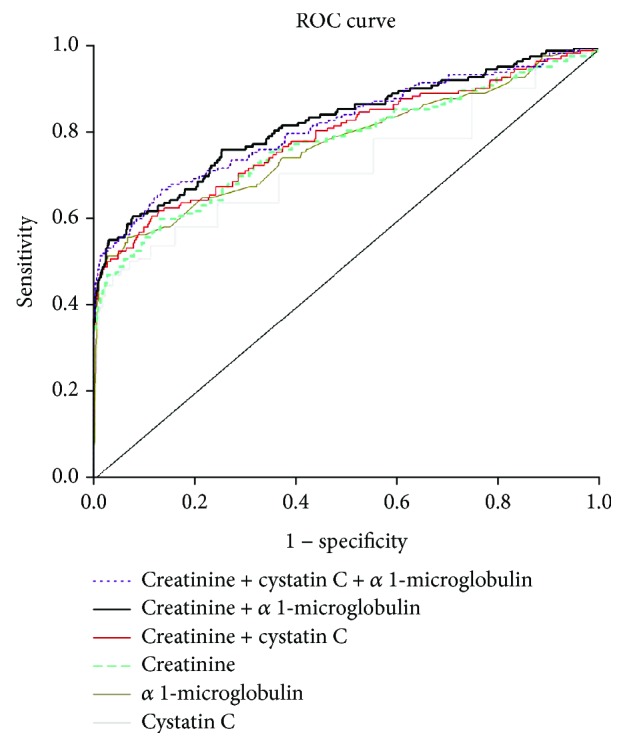


To compare the significance of multiple indicators (creatinine, cystatin C, and *α*_1_-MG) that had significant differences between the two groups in the identification of renal function, we have plotted ROC curves for these biomarkers (Figure 1). For the renal function biomarkers, there was no significant difference in the AUC for biomarkers (cystatin C, index: 0.728, CI 0.699-0.755; *α*_1_-MG: 0.775, CI 0.748-0.801; and cystatin C+creatinine: 0.794, CI 0.748-0.801) compared with creatinine. The AUC of combination of creatinine+*α*_1_-MG and creatinine+*α*_1_-MG+creatinine were statistically larger than those of creatinine, and the biomarker of the biggest AUC is the combination of creatinine+*α*_1_-MG (Table 6).

## 4. Discussion

There were 162 patients with renal involvement in this study, and the incidence rate was 16.17% (162/1002). In Goules's study, the prevalence of renal involvement was identified as 4.9% [6]. Another Chinese study also reported a relatively high incidence (33%) of renal abnormalities (based on biochemical abnormalities or kidney biopsy findings) in a study of 524 patients with PSS, 33% [7]. Because of a large number of study subjects in this work, our results suggest that the number of patients and geographical and ethnic factors might contribute to such variability.

PSS is characterized by B-cell activation with high serum IgG levels and a high frequency of autoantibodies [8]. In our study, pSS patients had multiple autoantibodies such as anti-SSA, anti-SSB, and ANA antibody, suggesting that pSS with renal abnormalities may be related to immune dysfunction. However, the pathological features of pSS with renal damage are the lymphocytic infiltration of the renal parenchyma rather than immune complex deposition and renal tubular atrophy that mainly presented interstitial nephritis mediated by an immune mechanism [9–11]. Although investigations about treatments targeting the immune factors participating in the progression of pSS show some positive outcome, more clinical trials were required before their application in human [12].

Among various manifestations of renal involvement, glomerular arterioles may be pathologically changed to glomerulonephritis, and a previous study showed that tubulointerstitial nephritis (TIN) is the most common presentation of renal involvement in the biopsy of pSS, which is consistent with our study [13].

Creatinine is primarily eliminated by glomerular filtration, and it can be used as a convenient means for estimating the glomerular filtration rate. Therefore, measurement of serum creatinine levels is the most common method used clinically for the routine monitoring of renal function [14]. Several studies have shown that serum cystatin C levels were more sensitive for detecting early and mild changes in renal function compared with the sensitivity of serum creatinine levels [15]. Serum cystatin C was produced at a constant rate by all nucleated body cells and was independent of age and gender [16–18]. Cystatin C was freely filtered at the glomerulus and was neither secreted nor reabsorbed by renal tubules [19]. Cystatin can reflect the decline of glomerular filtration rate that was the most direct indicator of renal damage, and it can be used as markers for early renal damage [20, 21]. In our study, the level of cystatin C showed a significant difference between patients with and without renal involvement and was identified as a potential risk factor for renal involvement, which was consistent with another study.

*α*_1_-MG was described and isolated from the urine of patients with chronic cadmium poisoning in 1975 [22]. The biochemical characteristics and clinical application value of alpha-1-microglobulin have been studied by scholars. It is synthesized not only by lymphocytes in the human body [2] but also by the liver [23], and it widely exists in various body fluids and on the surface of lymphocytes. *α*_1_-MG also is a stable urinary indicator protein which reflects acute and chronic dysfunctions of the proximal renal tubule. Our laboratory examination showed that the level of alpha-1-microglobulin in the pSS with renal damage group was significantly higher than that in the nonrenal damage group, which indicated the damage of proximal renal tubule and subsequent immune response to lymphocyte infiltration of the renal parenchyma in pSS. The combination of creatinine and *α*_1_-MG had the best AUC, indicating that the combination of creatinine and *α*_1_-MG was more effective in identifying renal function in pSS.

However, limitations of this study should be indicated. Firstly, the limited sample size, as well as bias caused by single-center analysis, should be considered, and secondly, as a cross-sectional study, it is limited to correlation analysis and unable to support strong causal conclusions. Therefore, to further evaluate the role of complement renal complications in SS, more data from heterogeneous SS patients with consecutive follow-up are highly recommended.

## 5. Conclusions

Renal involvement is common in pSS patients. The combination of creatinine and *α*_1_-MG is a better indicator of renal function for pSS patients, and close attention should be paid to it in clinical practice.

## Figures and Tables

**Table 1:** Demographic, clinical, histological, immunological, and inflammatory features of primary Sjogren's syndrome with or without renal involvement.

	Without renal involvement 840	With renal involvement 162	*P* value
Seroperitoneum	0	6 (3.7%)	0.000
Dizziness	12 (1.4%)	8 (4.9%)	0.003
Palpitate	14 (1.7%)	6 (3.7%)	0.090
Breathe hard	26 (3.1%)	9 (5.6%)	0.118
Digestive tract symptoms	19 (2.3%)	31 (19.1%)	0.000
Respiratory system symptoms	30 (3.6%)	15 (9.3%)	0.001
Congestion of throat	1 (0.1%)	10 (6.2%)	0.000
Bilateral pleural effusion	0	3 (1.9%)	0.000
Lipsotrichia	53 (6.3%)	4 (2.5%)	0.053
Dry cough	4 (0.5%)	1 (0.6%)	0.815
Edema in the face	5 (0.6%)	14 (8.6%)	0.000
Edema of both lower limbs	13 (1.5%)	42 (25.9%)	0.000
Hypourocrinia	0	4 (2.5%)	0.000
Frequent micturition	8 (1.0%)	10 (6.2%)	0.000
Urgency of urine	8 (1.0%)	7 (4.3%)	0.000
Odynuria	3 (0.4%)	4 (2.5%)	0.003
Rampant caries	187 (22.3%)	34 (21%)	0.720
Erythema	149 (17.7%)	23 (14.2%)	0.274
Weak	119 (14.2%)	50 (30.9%)	0.000
Poor appetite	9 (1.0%)	25 (15.4%)	0.000
Dry mouth	669 (79.6%)	129 (79.6%)	0.997
Xerophthalmia	457 (54.4%)	94 (58%)	0.396
Arthralgia	493 (58.7%)	68 (42%)	0.000
Fever	149 (17.7%)	28 (17.3%)	0.890
Reynolds	33 (3.9%)	9 (5.6%)	0.344
Dental ulcer	69 (8.2%)	16 (9.9%)	0.487
Courpature	1 (0.1%)	1 (0.6%)	0.193
Hematuria	0	1 (0.6%)	0.023
Polydipsia	1 (0.1%)	2 (1.2%)	0.021
Diuresis	1 (0.1%)	2 (1.2%)	0.021
Nocturia	2 (0.2%)	17 (18.5%)	0.000
Parotid swelling and pain	29 (3.5%)	3 (1.9%)	0.289
Anti-scl-70	0	2 (1.2%)	0.001
Anti-Jo-1	0	2 (1.2%)	0.001
pANCA	17 (2%)	4 (2.5%)	0.717
cANCA	3 (0.4%)	1 (0.6%)	0.631
RF	144 (17.1%)	44 (27.2%)	0.003
Anti-ENA	169 (20.1%)	59 (36.4%)	0.000
Anti-ds DNA	18 (2.1%)	3 (1.9%)	0.813
Anti-SSA	579 (68.9%)	80 (49.4%)	0.000
Anti-SSB	54 (6.4%)	19 (11.7%)	0.017
Anti-Sm	4 (0.5%)	4 (2.5%)	0.009
Anti-RNP	87 (10.4%)	15 (9.3%)	0.672

**Table 2:** Demographic, clinical, histological, immunological and inflammatory features of primary Sjogren's syndrome with or without renal involvement.

	Without renal involvement 840	With renal involvement 162	*P* value
Age	49.46 ± 13.36	49.94 ± 15.39	0.713
Mouth disease	69.44 ± 83.05	56.76 ± 91.78	0.082
White blood cell	6.15 ± 3.035	6.34 ± 3.03	0.485
RBC	4.08 ± 0.61	3.66 ± 0.87	0.000
Hb	122.61 ± 26.56	109.75 ± 24.94	0.000
Platelet	209.61 ± 101.51	214.66 ± 100.74	0.562
Monocyte	0.43 ± 0.25	0.45 ± 0.42	0.402
Eosinophil	0.11 ± 0.17	0.13 ± 0.16	0.181
Lymphocyte%	28.73 ± 11.31	28.57 ± 11.82	0.872
Lymphocyte	1.66 ± 1.64	1.66 ± 0.84	0.973
Monocyte%	7.44 ± 3.85	6.77 ± 2.49	0.005
Eosinophil%	1.86 ± 2.35	1.97 ± 2.15	0.568
Urine RBC	5.79 ± 38.71	26.95 ± 100.88	0.009
Urine WBC	18.30 ± 57.64	11.60 ± 39.55	0.071
Urine pH	6.34 ± 0.78	6.28 ± 1.00	0.463
Proportion	1.02 ± 0.01	1.02 ± 0.01	0.074
ALT	32.81 ± 35.05	30.9 ± 77.11	0.618
AST	32.66 ± 33.19	35.79 ± 80.43	0.626
AST/ALT	1.18 ± 0.56	1.28 ± 0.47	0.039
Total bilirubin	14.26 ± 14.24	13.04 ± 33.11	0.445
Direct bilirubin	4.19 ± 7.15	4.53 ± 19.00	0.822
Indirect bilirubin	10.06 ± 8.34	8.77 ± 14.62	0.121
Prealbumin	234.65 ± 56.94	262.90 ± 66.57	0.000
Total protein	71.10 ± 10.40	68.16 ± 11.80	0.000
Albumin	37.28 ± 5.51	34.475 ± 6.87	0.004
Globulin	33.45 ± 9.10	33.70 ± 9.48	0.000
Albumin/globulin	1.19 ± 0.35	1.10 ± 0.36	0.001
Alkaline phosphatase	99.26 ± 99.52	102.35 ± 68.45	0.687
Glutamyl transpeptidase	53.52 ± 102.20	37.64 ± 69.78	0.015
Total bile acid	9.73 ± 20.71	10.21 ± 37.32	0.815
5-Nucleoglykase	8.45 ± 14.37	6.73 ± 10.15	0.147
Adenosine deaminase	19.01 ± 11.93	18.84 ± 9.07	0.858
Blood glucose (4.2-6.1)	5.31 ± 1.57	5.18 ± 1.06	0.332
Fructosamine	1.83 ± 0.65	1.81 ± 0.56	0.633
Urea nitrogen	4.50 ± 1.76	8.43 ± 6.62	0.000
Creatinine	55.70 ± 14.32	150.82 ± 150.41	0.000
CO_2_ CP	25.08 ± 2.84	22.54 ± 4.60	0.000
Cystatin C	1.09 ± 0.36	2.04 ± 1.38	0.000
*α*_1_-MG (10-30 ng/L)	20.89 ± 7.95	34.04 ± 15.93	0.000
*β*_2_-MG (0.97-2.64 ng/L)	2.81 ± 5.19	6.02 ± 5.64	0.000
Uric acid (90-420 *μ*mol/L)	246.66 ± 78.60	292.70 ± 115.11	0.000
Complement-C1q (159-233 mg/L)	198.06 ± 14.16	199.79 ± 15.72	0.164
K (3.5-5.5 mmol/L)	3.91 ± 0.41	3.93 ± 0.63	0.631
Na (137-147 mmol/L)	139.50 ± 3.47	139.31 ± 4.08	0.579
Cl (99-110 mmol/L)	105.34 ± 3.66	107.38 ± 5.36	0.000
Ca (2.08-2.6 mmol/L)	2.24 ± 0.14	2.19 ± 0.18	0.000
P (0.83-1.48 mmol/L)	1.23 ± 0.50	1.26 ± 0.30	0.465
Mg (0.7-1.1 mmol/L)	0.91 ± 0.10	0.93 ± 0.12	0.145
Fe	14.07 ± 6.61	13.31 ± 7.20	0.190
CK	66.41 ± 124.01	82.60 ± 192.51	0.304
CK-MB	9.39 ± 8.96	8.90 ± 6.54	0.502
LDH	221.41 ± 168.21	233.16 ± 201.45	0.432
HBD	173.22 ± 141.13	180.03 ± 136.94	0.572
Total cholesterol	4.55 ± 1.25	4.53 ± 1.82	0.883
Triglyceride	1.90 ± 2.05	2.18 ± 2.36	0.156
HDL	1.21 ± 0.43	1.14 ± 0.37	0.054
LDL	2.66 ± 0.84	2.61 ± 1.25	0.607
Apolipoprotein-A_1_	1.30 ± 0.39	1.23 ± 0.32	0.033
Apolipoprotein-B100	0.84 ± 0.23	0.90 ± 0.38	0.581
Apolipoprotein-E	38.84 ± 12.74	40.23 ± 22.27	0.443
Lipoprotein-*α*	18.41 ± 18.30	23.19 ± 21.88	0.010
HDL/cholesterol	27.13 ± 7.68	21.51 ± 9.02	0.615
Acid phosphatase	4.29 ± 2.76	5.15 ± 2.82	0.000
ESR	38.29 ± 33.68	54.84 ± 36.36	0.000
CRP	10.35 ± 20.25	10.62 ± 20.50	0.877
Complement-C3	1.01 ± 0.24	0.95 ± 0.22	0.004
Complement-C4	0.23 ± 0.25	0.25 ± 0.14	0.370
PTH	38.52 ± 17.82	220.28 ± 307.65	0.032
CA19-9 (<35 KU/L)	12.45 ± 16.20	13.12 ± 15.81	0.624
CEA < 5 ng/L	2.17 ± 1.55	2.33 ± 0.99	0.021
AFP < 20 ng/L	2.81 ± 2.06	2.73 ± 2.36	0.655
IgG	14.84 ± 6.78	14.76 ± 7.59	0.891
IgA	2.86 ± 1.46	3.02 ± 1.31	0.192
IgM	1.64 ± 1.39	1.37 ± 0.75	0.000
Light chain quantitative *κ* (5.74-12.8 g/L)	7.87 ± 26.02	11.96± 8.27	0.283
Light chain quantitative L (2.69-6.38 g/L)	2.08 ± 3.87	86.07 ± 565.90	0.294

**Table 3:** Pathological types of kidney in 12 PSS patients with renal involvement.

Pathological type	Case
Mild mesangial proliferative nephritis with subacute tubulointerstitial nephropathy	1
Stage III glomerulosclerosis of nodular sclerosing diabetes mellitus	1
Mild mesangial hyperplasia	1
Atypical membranous nephropathy	1
Changes of renal tubular injury during convalescence	1
Focal proliferative sclerosing glomerulonephritis	1
Focal proliferative IgA nephropathy	1
Subacute tubulointerstitial nephropathy	1
Mild mesangial proliferative nephritis with subacute tubulointerstitial nephropathy	1
Stages I-II membranous nephropathy	1
Chronic interstitial renal damage	1
Atypical membranous nephropathy with multiple crescents and acute tubular injury	1

**Table 4:** Features of renal involvement in primary Sjogren's syndrome patients.

Renal involvement	Numbers	Percentage (%)
Edema in the face	14	8.6
Edema of both lower limbs	42	25.9
Hypourocrinia	4	2.5
Frequent micturition	10	6.2
Urgency of urine	7	4.3
Hematuria	1	0.6
Diuresis	2	1.2
Nocturia	17	18.5
Interstitial nephritis	6	3.7
Renal tubular acidosis	12	7.4

**Table 5:** Multivariate analysis of factors associated with renal involvement in primary Sjogren's syndrome.

Independent variables	Muitivariate analysis OR (95% Cl)	*P* value
Arthralgia	1.32 (0.79, 2.22)	0.294
Weak	1.83 (1.01, 3.31)	0.046
Poor appetite	1.52 (0.34, 6.74)	0.580
Edema in the face	3.33 (0.58, 19.25)	0.179
Edema of both lower limbs	9.16 (3.18, 26.39)	0.000
Hypourocrinia	3768741.41 (0.00)	0.999
Frequent micturition	2.30 (0.03, 197.13)	0.714
Urgency of urine	0.51 (0.01, 27.65)	0.740
Odynuria	1.46 (0.02, 87.33)	0.856
Hematuria	97021762.92 (0.00)	1.000
Polydipsia	2521.28 (0.00)	0.999
Diuresis	0.00 (0.00)	0.999
Digestive tract symptoms	3.06 (1.02, 9.22)	0.047
Respiratory system symptoms	0.83 (0.23, 3.01)	0.779
Congestion of throat	9.02 (0.16, 507.78)	0.285
Bilateral pleural effusion	16009499.05 (0.00)	0.999
RBC (3.5-5.5 × 10^12^/L)	1.12 (0.70, 1.81)	0.637
Hb (110-150 g/L)	1.00 (0.99, 1.01)	0.831
Urine RBC	1.01 (1.00, 1.01)	0.015
AST/ALT	1.00 (0.68, 1.49)	0.987
Prealbumin	1.01 (1.00, 1.01)	0.026
Total protein (65-85 g/L)	0.99 (0.95, 1.04)	0.778
A/G	1.37 (0.28, 6.68)	0.699
Creatinine (44-133 *μ*mol/L)	1.03 (1.01, 1.04)	0.000
Urea nitrogen (2.8-68.2 mmol/L)	0.97 (0.85, 1.10)	0.628
CO_2_ CP (22-29 mmol/L)	0.95 (0.87, 1.03)	0.220
Cystatin C (0.1-0.3 mmol/L)	1.83 (1.16, 2.87)	0.009
*α*_1_-MG (10-30 mg/L)	1.03 (1.00, 1.05)	0.021
Uric acid (90-420 *μ*mol/L)	1.00 (1.00, 1.00)	0.323
*β*_2_-MG (0.97-2.64 mg/L)	1.01 (0.96, 1.06)	0.805
Cl (99-110 mmol/L)	1.10 (1.03, 1.12)	0.004
Ca (2.08-2.6 mmol/L)	3.49 (0.47, 25.83)	0.221
Apolipoprotein A_1_	0.56 (0.26, 1.20)	0.134
Lipoprotein-*α*	1.00 (0.99, 1.02)	0.508
Acid phosphatase (1-9 U/L)	1.00 (0.91, 1.09)	0.916
ESR	1.01 (1.00, 1.02)	0.126
Complement-C3 (30.8-82.01 g/L)	0.46 (0.15, 1.37)	0.161
IgM	0.91 (0.71, 1.16)	0.434

**Table 6:** AUC of creatinine, cystatin C, and *α*_1_-MG.

	AUC	95% CI	*P* value
Creatinine	0.777	0.750-0.803	
Cystatin C	0.728	0.699-0.755	>0.05 (vs. creatinine)
*α*1-Microglobulin	0.775	0.748-0.801	>0.05 (vs. creatinine)
Creatinine+cystatin C	0.794	0.767-0.819	>0.05 (vs. creatinine)
Creatinine+*α*1-microglobulin	0.824	0.799-0.847	<0.05 (vs. creatinine)
Creatinine+cystatin C + *α*1-microglobulin	0.819	0.794-0.843	<0.05 (vs. creatinine)

AUC: area under the curve; CI: confidence interval.

## Data Availability

The data used to support the findings of this study are available from the corresponding author upon request.
